# An Intragenic Recombination Event Generates a Snf4-Independent Form of the Essential Protein Kinase Snf1 in Candida albicans

**DOI:** 10.1128/mSphere.00352-19

**Published:** 2019-06-19

**Authors:** Austin Mottola, Joachim Morschhäuser

**Affiliations:** a Institut für Molekulare Infektionsbiologie, Universität Würzburg, Würzburg, Germany; Carnegie Mellon University

**Keywords:** AMP-activated kinases, *Candida albicans*, genetic recombination, metabolic adaptation, suppressor mutation

## Abstract

The heterotrimeric protein kinase SNF1 plays a key role in the metabolic adaptation of the pathogenic yeast Candida albicans. It consists of the essential catalytic α-subunit Snf1, the γ-subunit Snf4, and one of the two β-subunits Kis1 and Kis2. Snf4 is required to release the N-terminal catalytic domain of Snf1 from autoinhibition by the C-terminal regulatory domain, and *snf4*Δ mutants cannot grow on carbon sources other than glucose. In a screen for suppressor mutations that restore growth of a *snf4*Δ mutant on alternative carbon sources, we isolated a mutant in which six amino acids between the N-terminal kinase domain and the C-terminal regulatory domain of Snf1 were deleted. The deletion was caused by an intragenic recombination event between two 8-bp direct repeats flanking six intervening codons. In contrast to truncated forms of Snf1 that contain only the kinase domain, the Snf4-independent Snf1^Δ311 − 316^ was fully functional and could replace wild-type Snf1 for normal growth, because it retained the ability to interact with the Kis1 and Kis2 β-subunits via its C-terminal domain. Indeed, the Snf4-independent Snf1^Δ311 − 316^ still required the β-subunits of the SNF1 complex to perform its functions and did not rescue the growth defects of *kis1*Δ mutants. Our results demonstrate that a preprogrammed in-frame deletion event within the *SNF1* coding region can generate a mutated form of this essential kinase which abolishes autoinhibition and thereby overcomes growth deficiencies caused by a defect in the γ-subunit Snf4.

**IMPORTANCE** Genomic alterations, including different types of recombination events, facilitate the generation of genetically altered variants and enable the pathogenic yeast Candida albicans to adapt to stressful conditions encountered in its human host. Here, we show that a specific recombination event between two 8-bp direct repeats within the coding sequence of the *SNF1* gene results in the deletion of six amino acids between the N-terminal kinase domain and the C-terminal regulatory domain and relieves this essential kinase from autoinhibition. This preprogrammed deletion allowed C. albicans to overcome growth defects caused by the absence of the regulatory subunit Snf4 and represents a built-in mechanism for the generation of a Snf4-independent Snf1 kinase.

## Abstract (precis)

Genomic alterations, including different types of recombination events, facilitate the generation of genetically altered variants and enable the pathogenic yeast Candida albicans to adapt to stressful conditions encountered in its human host. Here, we show that a specific recombination event between two 8-bp direct repeats within the coding sequence of the *SNF1* gene results in the deletion of six amino acids between the N-terminal kinase domain and the C-terminal regulatory domain and relieves this essential kinase from autoinhibition. This preprogrammed deletion allowed C. albicans to overcome growth defects caused by the absence of the regulatory subunit Snf4 and represents a built-in mechanism for the generation of a Snf4-independent Snf1 kinase.

## INTRODUCTION

The metabolic flexibility of the opportunistic fungal pathogen Candida albicans is important for its ability to colonize and infect different body locations in its human host (1–3). Many host niches contain only low levels of glucose, which is the preferred carbon source, and the ability to utilize alternative carbon sources is critical for the virulence of C. albicans (1, 4–6). A key regulator of the adaptation to glucose limitation and utilization of alternative carbon sources is the heterotrimeric protein kinase SNF1, a member of the AMP-activated protein kinase (AMPK) family, which is highly conserved in eukaryotic organisms and has been studied in great detail in the model yeast Saccharomyces cerevisiae (7). In S. cerevisiae, the SNF1 complex consists of the catalytic α-subunit Snf1, the γ-subunit Snf4, and one of three alternative β-subunits, Sip1, Sip2, and Gal83. Snf4 binds to the C-terminal regulatory domain of Snf1, thereby releasing the N-terminal catalytic domain from autoinhibition under inducing conditions (8). The β-subunits, which also bind to a C-terminal region in Snf1, are responsible for the interaction with target proteins and regulate the subcellular localization of the kinase (9–11). Snf1 is activated in response to glucose limitation and other stresses via phosphorylation at Thr210 in its activation loop by the three partially redundant upstream activating kinases Elm1, Sak1, and Tos3 (12–14). It is dephosphorylated at Thr210 and thereby inactivated by Reg1-Glc7 protein phosphatase 1 (15, 16). In C. albicans, the SNF1 complex consists of the α-subunit Snf1, the γ-subunit Snf4, and one of the two β-subunits Kis1 and Kis2 (17).

Despite the structural and functional conservation of the SNF1 complex, there are some notable differences between S. cerevisiae and C. albicans. In C. albicans, Snf1 is constitutively phosphorylated at Thr208 (corresponding to Thr210 of ScSnf1) in the activation loop (18), and this phosphorylation is mediated by a single upstream activating kinase, Sak1 (19). Remarkably, while S. cerevisiae *snf1*Δ mutants are viable, *SNF1* seems to be an essential gene in C. albicans (19–22). In contrast, the genes encoding the upstream kinase Sak1 or the β-subunits Kis1 and Kis2 and the γ-subunit Snf4 of the SNF1 complex can be deleted. C. albicans *sak1*Δ and *snf4*Δ mutants have growth defects on alternative carbon sources and are highly sensitive to cell wall/membrane stress but are viable (19). This observation indicates that Snf1 retains some basal function that is essential for viability even in its unphosphorylated state or when other subunits of the SNF1 complex are absent.

Although the function of Snf1 in C. albicans is similar to that of its homologs in S. cerevisiae and other organisms, it has not been established how the kinase regulates metabolic activities in this pathogenic yeast. Several cases of transcriptional rewiring of metabolic pathways have been described where the expression of functionally related genes is controlled by different transcription factors in S. cerevisiae and C. albicans (23–25). In C. albicans, the transcriptional repressor Mig1, whose activity is regulated by Snf1-dependent phosphorylation in S. cerevisiae, does not contain a consensus recognition sequence for Snf1, suggesting that the target proteins of Snf1 might differ in the two species (26). Insight into how the Snf1 kinase enables metabolic adaptation of C. albicans might be gained from the isolation of suppressor mutations that restore growth in mutants with a defective SNF1 complex. We have used this approach with *snf4*Δ mutants, which (in contrast to *snf1*Δ mutants) are viable but have stronger growth defects than *sak1*Δ mutants and cannot grow at all on alternative carbon sources. Here, we report the isolation and characterization of a suppressor mutation in the Snf1 kinase itself, which arose by an intragenic recombination event that resulted in the deletion of 6 internal amino acids and rendered the SNF1 complex functional in the absence of the γ-subunit Snf4.

## RESULTS

### An in-frame deletion in *SNF1* suppresses *snf4*Δ mutant phenotypes.

**FIG 1:**
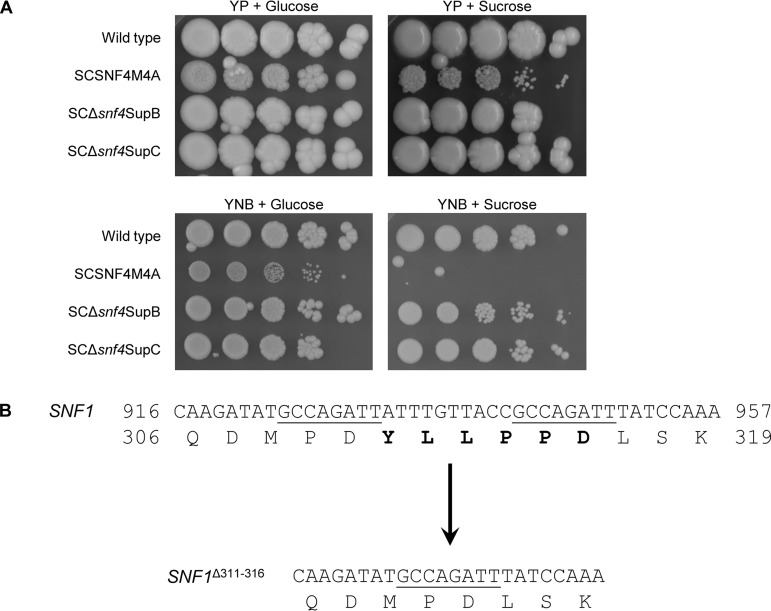
An in-frame deletion in *SNF1* suppresses the growth defect of a *snf4*Δ mutant on sucrose as the sole carbon source. (A) Growth of wild-type strain SC5314, *snf4*Δ mutant SCSNF4M4A, and two spontaneous suppressor mutants (SCΔ*snf4*SupB and SCΔ*snf4*SupC) on YP agar plates (top panels) and YNB agar plates (bottom panels) containing 2% glucose or 2% sucrose as the carbon source. YPD overnight cultures of the strains were adjusted to an optical density (OD_600_) of 2.0 and serial 10-fold dilutions plated and incubated for 4 days at 30°C. (B) Partial nucleotide and deduced amino acid sequences of the *SNF1* alleles of wild-type strain SC5314 (top; positions within the *SNF1* coding sequence and Snf1 protein sequence are indicated) and of the mutated *SNF1* allele of suppressor mutant SCΔ*snf4*SupB (bottom). The 8-bp direct repeat sequence in the wild-type allele is underlined, and the amino acids deleted in Snf1^Δ311 − 316^ are highlighted in bold.

C. albicans mutants lacking the γ-subunit Snf4 of the SNF1 complex are unable to grow on alternative carbon sources and also exhibit reduced growth on glucose (19). We hypothesized that cultivation of *snf4*Δ mutants in glucose-containing media would enrich for faster-growing spontaneous mutants and allow the isolation of suppressor mutations that bypass the dependence on Snf4 for growth on alternative carbon sources. Therefore, a *snf4*Δ mutant was passaged twice in yeast extract-peptone-dextrose (YPD) medium, and the cultures were plated on YNB minimal medium containing sucrose as the sole carbon source. By this procedure, we were able to isolate two mutants, designated SCΔ*snf4*SupB and SCΔ*snf4*SupC, which appeared as single colonies on YNB + sucrose plates after the first and second YPD subcultures, respectively. Dilution spot assays confirmed that both suppressor mutants had regained the ability to utilize sucrose despite the absence of Snf4 and also exhibited improved growth on glucose (Fig. 1A).

A candidate for *snf4*Δ suppressor mutations is the gene encoding the catalytic α-subunit Snf1 of the SNF1 complex, because mutations in Snf1 that reduce its dependence on Snf4 have been identified in S. cerevisiae (27–29). We therefore amplified the *SNF1* alleles of the suppressor mutants and directly sequenced the PCR products. The two *SNF1* alleles of C. albicans strain SC5314, the wild-type parent of our mutants, differ slightly. In comparison with allele A (the reference sequence), allele B lacks one of the 10 histidine codons in the N-terminal polyhistidine tract and contains the silent nucleotide exchanges A1443G, T1620C, and A1809G (http://www.candidagenome.org). No mutations were found in the *SNF1* alleles of strain SCΔ*snf4*SupC, and the basis of the suppressor phenotype of this strain will be the subject of future investigations. In contrast, strain SCΔ*snf4*SupB contained an in-frame deletion of codons 311 to 316 in one of the *SNF1* alleles. As illustrated in Fig. 1B, this deletion most likely occurred by an intragenic recombination event between two 8-bp direct repeats bordering the deleted region. Using primers that bind within the deleted sequence, we determined that the deletion had occurred in allele A whereas allele B was unaltered.

**FIG 2:**
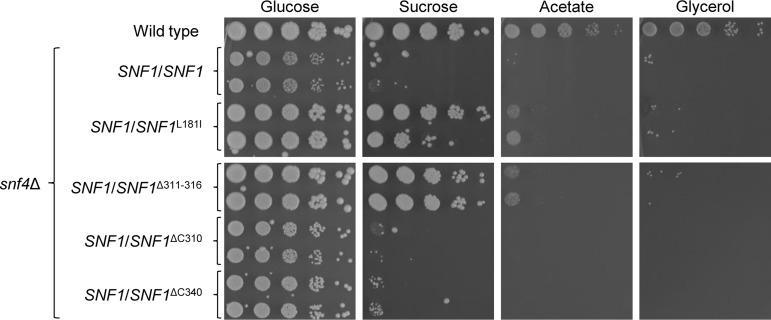
Growth of wild-type strain SC5314, *snf4*Δ mutants (SCSNF4M4A and SCSNF4M4B), and derivatives in which one endogenous *SNF1* allele was replaced by the indicated mutated allele on different carbon sources. YPD overnight cultures of the strains were adjusted to an optical density (OD_600_) of 2.0 and serial 10-fold dilutions spotted on YNB agar plates containing 2% glucose, sucrose, acetate, or glycerol as the sole carbon source. Plates were incubated for 4 days at 30°C. Both independently generated series of mutants are shown. Strains in the top and bottom panels were grown on the same plate, and the photographs are arranged accordingly for clarity of presentation.

To verify that the deletion of six amino acids within Snf1 was the reason for the improved growth of strain SCΔ*snf4*SupB, we introduced the same 18-bp deletion into one of the endogenous *SNF1* alleles in two independently generated *snf4*Δ mutants. For comparison, we also introduced an L181I mutation, which corresponds to an L183I *snf4*Δ suppressor mutation in Snf1 of S. cerevisiae (29) and which we had previously found to partially suppress the growth defects of *sak1*Δ mutants (19), into the *snf4*Δ mutants. As illustrated in Fig. 2, the two mutations were equally efficient in restoring growth of cells lacking Snf4 on different carbon sources. Growth on glucose was improved, and growth on sucrose was also restored to nearly wild-type levels. The growth defect on acetate was partially rescued, while no growth improvement on glycerol was observable. These results show that the deletion of amino acids 311 to 316 in Snf1 rendered the protein partially independent of the presence of the regulatory γ-subunit Snf4.

In a previous study, we had found that a C-terminally truncated Snf1 containing only the first 340 amino acids, which corresponds to a hyperactive form of mammalian AMPK, did not remediate the growth defects of C. albicans *snf4*Δ mutants (19). Interestingly, the internal deletion in Snf^Δ311 − 316^ is located near the end of a C-terminally truncated Snf1 of S. cerevisiae that exhibited reduced dependence on Snf4 (27). We therefore hypothesized that a complete C-terminal truncation of Snf1 after amino acid 310 instead of amino acid 340, resulting in a protein that contains only the kinase domain (amino acids 53 to 304), might also suppress *snf4*Δ mutant phenotypes. However, similarly to *SNF1*^ΔC340^, the *SNF1*^ΔC310^ allele did not improve growth of the *snf4*Δ mutants (Fig. 2). This may be due to the fact that the C-terminal truncations removed not only the autoinhibitory domain of Snf1 but also the interaction domain with β-subunits Kis1 and Kis2, thereby impairing the functionality of the kinase, in line with results previously obtained in S. cerevisiae (29). In contrast, the internal deletion of only six amino acids directly behind the kinase domain probably abolished its interaction with the inhibitory domain, such that Snf4 was no longer required to prevent this interaction, while it may still allow binding of Kis1 and Kis2 to Snf1 and formation of a functional SNF1 complex.

### Homozygosity augments the suppressor activity of mutated *SNF1* alleles.

**FIG 3:**
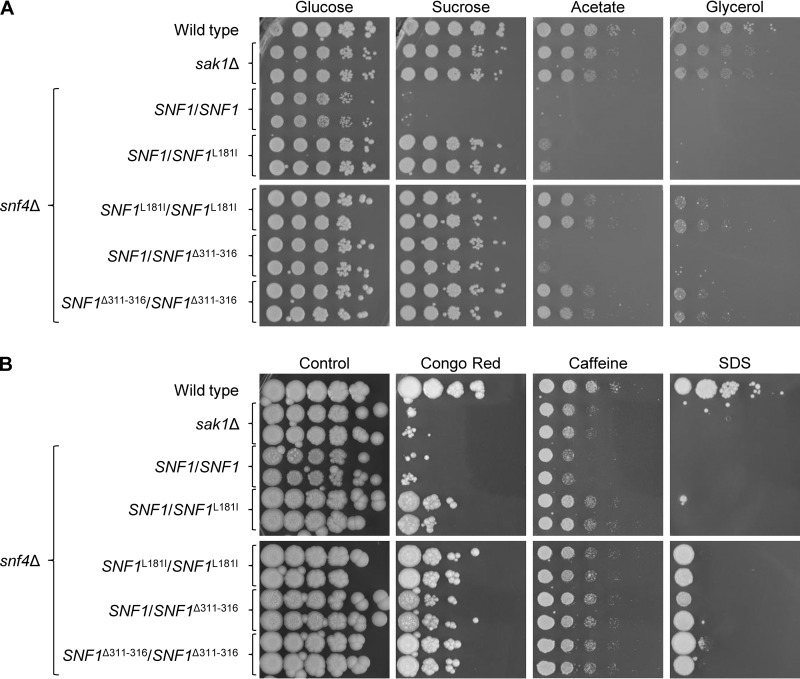
Growth of wild-type strain SC5314, *sak1*Δ mutants (SC3840M4A and SC3840M4B), *snf4*Δ mutants (SCSNF4M4A and SCSNF4M4B), and derivatives in which one or both endogenous *SNF1* alleles were replaced by the *SNF1*^L181I^ or the *SNF1*^Δ311 − 316^ allele on different carbon sources and under various stress conditions. YPD overnight cultures of the strains were adjusted to an optical density (OD_600_) of 2.0 and serial 10-fold dilutions spotted on YNB agar plates containing the indicated carbon sources (A) or on YPD plates with the indicated stressors (B). Plates were incubated for 4 days at 30°C. Both independently generated series of mutants are shown. Strains in the top and bottom panels were grown on the same plate, and the photographs arranged accordingly for clarity of presentation.

Gain-of-function mutations in several transcription factors of C. albicans that confer increased fluconazole resistance have a stronger effect when they occur in both alleles of the respective genes (30). We therefore reasoned that the growth of *snf4*Δ mutants would be further improved if they were homozygous for suppressor mutations in *SNF1*. To test this possibility, we introduced the Δ311 − 316 and L181I mutations into both endogenous *SNF1* alleles of the *snf4*Δ mutants and compared the growth of heterozygous and homozygous strains on different carbon sources (Fig. 3A). Indeed, the two suppressor mutations further augmented growth on acetate in the homozygous strains, and growth on glycerol was now also partially restored. These phenotypes were similar to those of *sak1*Δ mutants lacking the upstream Snf1-activating kinase, which have weaker growth defects than *snf4*Δ mutants.

### The *SNF1*^Δ311 − 316^ allele suppresses other *snf4*Δ mutant phenotypes.

C. albicans *sak1*Δ and *snf4*Δ mutants are also hypersensitive to cell wall/membrane stress (19). We therefore tested if the suppressor mutations in Snf1 could increase the resistance of *snf4*Δ mutants to agents causing cell wall/membrane stress. As can be seen in Fig. 3B, both mutated forms of Snf1 improved the growth of *snf4*Δ mutants on plates containing Congo red, caffeine, or SDS. Although resistance to Congo red and SDS was not restored to wild-type levels, the suppressor mutants grew better in the presence of these inhibitors than *sak1*Δ mutants lacking the upstream activating kinase Sak1. These results demonstrate that the deletion of amino acids 311 to 316 in Snf1, like the L181I mutation, partially restores different functions of the SNF1 complex in the absence of the regulatory γ-subunit.

### The Δ311 − 316 mutation does not increase Snf1 phosphorylation.

**FIG 4:**
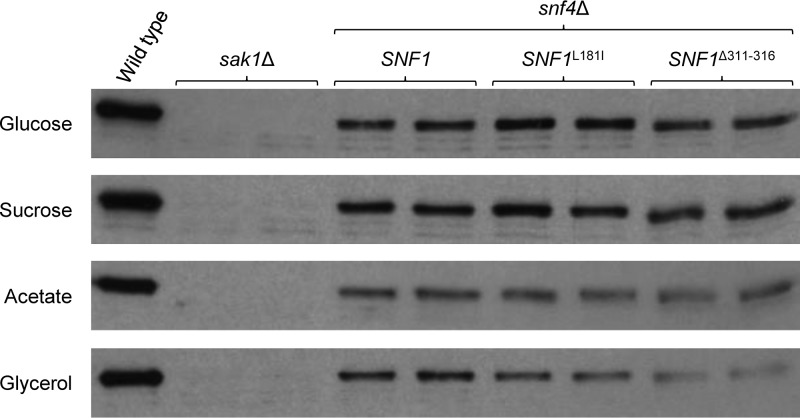
Thr208 phosphorylation of Snf1 in wild-type strain SC5314, *sak1*Δ mutants (SC3840M4A and SC3840M4B), *snf4*Δ mutants (SCSNF4M4A and SCSNF4M4B), and derivatives in which both endogenous *SNF1* alleles were replaced by the *SNF1*^L181I^ or the *SNF1*^Δ311 − 316^ allele. Protein extracts were prepared from cells grown in YP medium with glucose, sucrose, acetate, or glycerol and analyzed by Western blotting with an antibody against Thr208-phosphorylated Snf1. Both independently generated series of mutants are shown in each case.

In C. albicans mutants lacking the γ-subunit Snf4 of the SNF1 complex, Snf1 is still phosphorylated at Thr208 by the upstream activating kinase Sak1, but with lower efficiency, which may contribute to the growth defects of *snf4*Δ mutants (19). We therefore tested if the L181I and Δ311 − 316 mutations resulted in improved Snf1 phosphorylation in the absence of Snf4. However, no increased Snf1 phosphorylation was observed in *snf4*Δ mutants containing the suppressor mutations in all tested growth media (Fig. 4). In a previous study, we had observed that the L181I mutation also relieved the growth defects of *sak1*Δ mutants, in which Snf1 phosphorylation is abolished (19). Taken together, these results support the idea that the suppressor mutations partially restore the functionality of Snf1 in the absence of Snf4 by preventing autoinhibition independently of the Snf1 phosphorylation status.

### Snf1^Δ311 − 316^ activity requires the β-subunits of the SNF1 complex.

**FIG 5:**
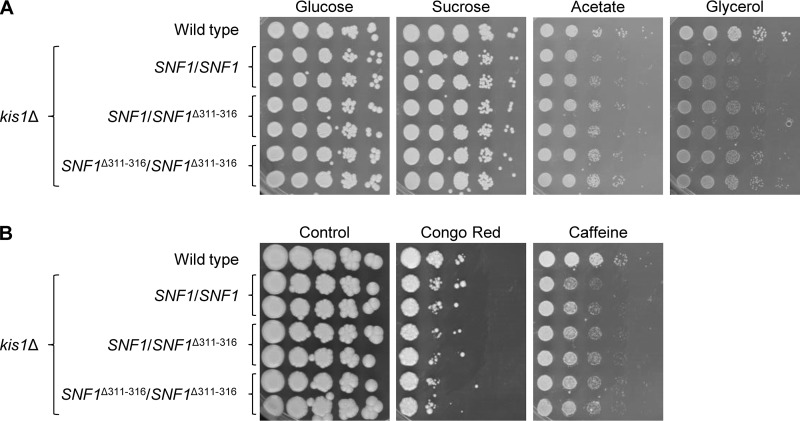
Growth of wild-type strain SC5314, *kis1*Δ mutants (SCKIS1M4A and SCKIS1M4B), and derivatives in which one or both endogenous *SNF1* alleles were replaced by the *SNF1*^Δ311 − 316^ allele on different carbon sources and under various stress conditions. YPD overnight cultures of the strains were adjusted to an optical density (OD_600_) of 2.0 and serial 10-fold dilutions spotted on YNB agar plates containing the indicated carbon sources (A) or on YPD plates with the indicated stressors (B). Plates were incubated for 4 days at 30°C. Both independently generated series of mutants are shown.

As discussed above, the ability of the *SNF1*^Δ311 − 316^ allele, but not the C-terminally truncated *SNF1*^ΔC310^ allele, to rescue growth defects of a *snf4*Δ mutant might have been due to functional interactions with the β-subunits Kis1 and Kis2. Mutants lacking Kis1 have growth defects on alternative carbon sources similar to those of *sak1*Δ mutants, while *kis2*Δ mutants grow as well as the wild type under these conditions. We therefore tested if the *SNF1*^Δ311 − 316^ allele could revert *kis1*Δ mutant phenotypes. As can be seen in Fig. 5, replacement of one or both wild-type *SNF1* alleles in *kis1*Δ mutants by the *SNF1*^Δ311 − 316^ allele did not markedly improve the growth of the mutants on alternative carbon sources or in the presence of cell wall stress. These results demonstrate that the Snf4-independent form of Snf1 still requires the β-subunits of the SNF1 complex to perform its functions.

### Functionality of Snf1^Δ311 − 316^ in a wild-type background.

**FIG 6:**
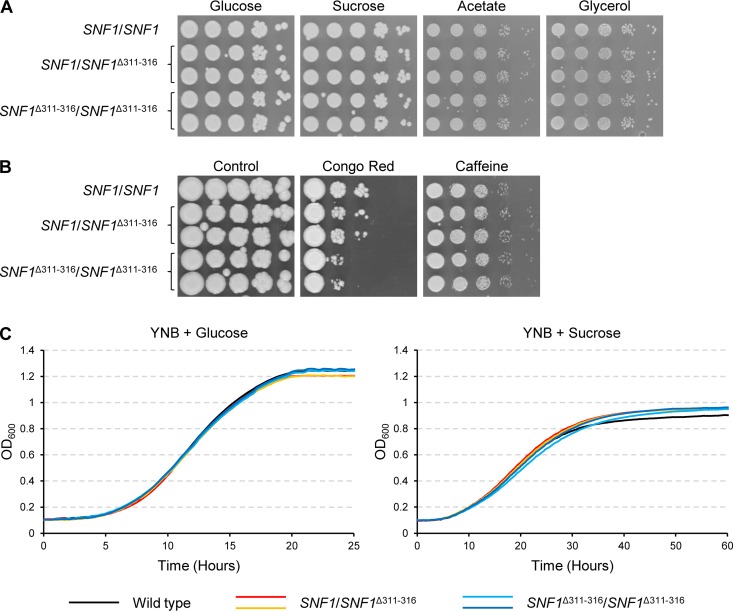
Growth of wild-type strain SC5314 and derivatives in which one or both endogenous *SNF1* alleles were replaced by the *SNF1*^Δ311 − 316^ allele on different carbon sources and under various stress conditions. Both independently generated series of mutants are shown. (A and B) YPD overnight cultures of the strains were adjusted to an optical density (OD_600_) of 2.0 and serial 10-fold dilutions spotted on YNB agar plates containing the indicated carbon sources (A) or on YPD plates with the indicated stressors (B). Plates were incubated for 4 days at 30°C. (C) Cells grown overnight in YNB + glucose were washed in water and diluted at an OD_600_ of 0.1 in fresh YNB + glucose or YNB + sucrose in a 96-well microtiter plate. Growth curves were obtained by measuring the OD of the cultures every 10 min in a Tecan Infinite F200 PRO plate reader. Curves represent averages of results from three biological replicates.

Finally, we investigated if the Δ311 − 316 mutation only diminished the dependence of Snf1 on Snf4 or if it resulted in a hyperactive form of the kinase. With this aim, we replaced one or both endogenous *SNF1* alleles by the *SNF1*^Δ311 − 316^ allele in the wild-type strain SC5314. As can be seen in Fig. 6A and B, the homozygous mutants grew as well as the wild-type strain under various tested conditions, except for a slight hypersusceptibility to Congo red, indicating that the internal deletion had little effect on the functionality of Snf1.

We reasoned that cells containing a hyperactive Snf1 might more rapidly adapt to changed growth conditions than wild-type cells and might exhibit a shorter lag phase after a switch from glucose to an alternative carbon source. To test if Snf1^Δ311 − 316^ provides such a growth advantage, overnight cultures of the strains grown in YNB + glucose medium were inoculated into fresh YNB medium with glucose or sucrose as the carbon source and growth was monitored by measuring the optical densities of the cultures over time. As can be seen in Fig. 6C, all strains grew more slowly on sucrose than on glucose, but no differences were observed between the wild type and the mutants in either medium, indicating that the mutated Snf1 did not enable the cells to adapt more quickly to the change in the carbon source.

## DISCUSSION

In this study, we identified a novel type of mutation in Snf1 that restores the functionality of the kinase in the absence of the normally required γ-subunit Snf4. Studies in S. cerevisiae have shown that Snf4 binds to the C-terminal part of Snf1 and (under inducing conditions) relieves the N-terminal catalytic domain from inhibition by the autoinhibitory domain (8). Screenings for suppressor mutations have revealed various point mutations in the catalytic domain that render Snf1 function independent of Snf4, indicating that autoinhibition is abolished in the mutated proteins (27–29). Unlike these mutations, the *snf4*Δ suppressor mutation described here is not located within the catalytic domain of Snf1; instead, the mutation resulted in the deletion of six amino acids between the N-terminal and C-terminal domains of the kinase. Internal deletions that bypass the requirement for Snf4 have also been engineered in Snf1 of S. cerevisiae; however, these deletions destroyed the autoinhibitory domain (29). In contrast, the small internal deletion of amino acids 311 to 316 directly behind the kinase domain left the inhibitory domain intact but most likely prevented its interaction with the kinase domain, thereby bypassing the dependence of Snf1 functionality on Snf4. The retention of the C-terminal domain enabled Snf1^Δ311 − 316^ to exert its normal functions in C. albicans, in contrast to the C-terminally truncated versions, which cannot interact with the β-subunits Kis1 and Kis2. Indeed, Snf1^Δ311 − 316^ still depended on the presence of the β-subunits to enable growth on alternative carbon sources, because it could not rescue the growth defect of *kis1*Δ mutants. The functions of Kis1 and Kis2 in C. albicans seem to be partially overlapping, as mutants lacking only one of them have comparatively mild growth defects (19). So far, we were unable to construct double mutants lacking both *KIS1* and *KIS2* (A. Mottola and J. Morschhäuser, unpublished results), suggesting that C. albicans requires at least one of the two β-subunits of the SNF1 complex for growth.

Another interesting aspect of our study is the mechanism by which the mutated *SNF1* allele arose. Unlike previously isolated suppressor mutations in *SNF1* of S. cerevisiae, which were point mutations resulting in amino acid exchanges or C-terminal deletions (27–29), the deletion of six codons in *SNF1*^Δ311 − 316^ occurred by a recombination event involving two flanking direct repeats. In contrast to point mutations, such a deletion is not a totally random event but can be regarded as a built-in mechanism that could involve recombination between the two repeats of one allele or unequal reciprocal recombination between the two homologous alleles, the latter resulting in hybrid alleles. The fact that allele-specific polymorphisms were retained on both sides of the deleted region in the mutated allele A and in the homologous wild-type allele B indicates that the deletion had occurred by an intra-allelic recombination event in our mutant.

As in other organisms, nucleotide sequence repeats are a rich source of genetic variation in C. albicans. In protein-encoding genes, these are usually tandem repeats that allow rapid expansion and shortening of amino acid repeat tracts, which may modify the functionality of the proteins (31). In contrast, the two 8-bp repeats in *SNF1* flank an intervening sequence, and recombination between these repeats leads to a precise in-frame deletion of six codons. In principle, the result of this recombination event is an irreversible deletion, which at first sight makes it unclear how the retention of the ability to undergo this event should be selected for. However, since C. albicans is a diploid organism, heterozygous mutants could revert to the original state by gene conversion using the wild-type allele as the template. In addition, even homozygous mutants might regain a wild-type *SNF1* allele by mating. Whether recombination between the two 8-bp repeats in *SNF1* is indeed used by C. albicans as a mechanism to generate a mutated form of Snf1 remains speculative. The experiments that we performed so far did not provide evidence that a Snf4-independent form of Snf1 would confer an advantage also in a wild-type background, but this might possibly be the case under some conditions encountered within the human host. In any case, the generation of a mutated form of *SNF1* by a preprogrammed intragenic recombination event is a new example of how the genomic flexibility of C. albicans can generate phenotypic variation and adaptation.

## MATERIALS AND METHODS

### Strains and growth conditions.

The C. albicans strains used in this study are listed in Table 1. All strains were stored as frozen stocks with 17.2% glycerol at −80°C and subcultured on YPD agar plates (10 g yeast extract, 20 g peptone, 20 g glucose, 15 g agar per liter) at 30°C. Strains were routinely grown in YPD liquid medium at 30°C in a shaking incubator. For selection of nourseothricin-resistant transformants, 200 μg/ml nourseothricin (Werner Bioagents, Jena, Germany) was added to YPD agar plates. To obtain nourseothricin-sensitive derivatives in which the *SAT1* flipper cassette was excised by FLP-mediated recombination, transformants were grown overnight in YCB-BSA-YE medium (23.4 g yeast carbon base [YCB], 4 g bovine serum albumin [BSA], 2 g yeast extract [YE] per liter, pH 4.0) without selective pressure to induce the *SAP2* promoter controlling *caFLP* expression. Appropriate dilutions were plated on YPD agar plates and grown for 2 days at 30°C. Individual colonies were picked and streaked on YPD plates as well as on YPD plates with 100 μg/ml nourseothricin to confirm nourseothricin sensitivity.

**TABLE 1:** C. albicans strains used in this study

Strain	Parent	Relevant characteristics or genotype^*a*^	Reference or source
SC5314		Wild-type reference strain	34
SC3840M4A and -B	SC5314	*sak1*Δ::*FRT*/*sak1*Δ::*FRT*	19
SCSNF4M4A and -B	SC5314	*snf4*Δ::*FRT*/*snf4*Δ::*FRT*	19
SCKIS1M4A and -B	SC5314	*kis1*Δ::*FRT*/*kis1*Δ::*FRT*	19
SCΔ*snf4*SupB	SCSNF4M4A	*snf4*Δ::*FRT*/*snf4*Δ::*FRT SNF1*/*SNF1*^Δ311 − 316^	This study
SCΔ*snf4*SupC	SCSNF4M4A	*snf4*Δ::*FRT*/*snf4*Δ::*FRT* (contains unknown suppressor mutation)	This study
SCΔ*snf4*SNF1^Δ311 − 316^M1A	SCSNF4M4A	*snf4*Δ::*FRT*/*snf4*Δ::*FRT SNF1*/*SNF1*^Δ311 − 316^*-SAT1-FLIP*	This study
SCΔ*snf4*SNF1^Δ311 − 316^M1B	SCSNF4M4B	*snf4*Δ::*FRT*/*snf4*Δ::*FRT SNF1*/*SNF1*^Δ311 − 316^*-SAT1-FLIP*	This study
SCΔ*snf4*SNF1^Δ311 − 316^M2A	SCΔ*snf4*SNF1^Δ311 − 316^M1A	*snf4*Δ::*FRT*/*snf4*Δ::*FRT SNF1*/*SNF1*^Δ311 − 316^*-FRT*	This study
SCΔ*snf4*SNF1^Δ311 − 316^M2B	SCΔ*snf4*SNF1^Δ311 − 316^M1B	*snf4*Δ::*FRT*/*snf4*Δ::*FRT SNF1*/*SNF1*^Δ311 − 316^*-FRT*	This study
SCΔ*snf4*SNF1^Δ311 − 316^M3A	SCΔ*snf4*SNF1^Δ311 − 316^M2A	*snf4*Δ::*FRT*/*snf4*Δ::*FRT SNF1*^Δ311 − 316^*-SAT1-FLIP*/*SNF1*^Δ311 − 316^*-FRT*	This study
SCΔ*snf4*SNF1^Δ311 − 316^M3B	SCΔ*snf4*SNF1^Δ311 − 316^M2B	*snf4*Δ::*FRT*/*snf4*Δ::*FRT SNF1*^Δ311 − 316^*-SAT1-FLIP*/*SNF1*^Δ311 − 316^*-FRT*	This study
SCΔ*snf4*SNF1^Δ311 − 316^M4A	SCΔ*snf4*SNF1^Δ311 − 316^M3A	*snf4*Δ::*FRT*/*snf4*Δ::*FRT SNF1*^Δ311 − 316^*-FRT*/*SNF1*^Δ311 − 316^*-FRT*	This study
SCΔ*snf4*SNF1^Δ311 − 316^M4B	SCΔ*snf4*SNF1^Δ311 − 316^M3B	*snf4*Δ::*FRT*/*snf4*Δ::*FRT SNF1*^Δ311 − 316^*-FRT*/*SNF1*^Δ311 − 316^*-FRT*	This study
SCΔ*snf4*SNF1^L181I^M1A	SCSNF4M4A	*snf4*Δ::*FRT*/*snf4*Δ::*FRT SNF1*/*SNF1*^L181I^*-SAT1-FLIP*	This study
SCΔ*snf4*SNF1^L181I^M1B	SCSNF4M4B	*snf4*Δ::*FRT*/*snf4*Δ::*FRT SNF1*/*SNF1*^L181I^*-SAT1-FLIP*	This study
SCΔ*snf4*SNF1^L181I^M2A	SCΔ*snf4*SNF1^L181I^M1A	*snf4*Δ::*FRT*/*snf4*Δ::*FRT SNF1*/*SNF1*^L181I^*-FRT*	This study
SCΔ*snf4*SNF1^L181I^M2B	SCΔ*snf4*SNF1^L181I^M1B	*snf4*Δ::*FRT*/*snf4*Δ::*FRT SNF1*/*SNF1*^L181I^*-FRT*	This study
SCΔ*snf4*SNF1^L181I^M3A	SCΔ*snf4*SNF1^L181I^M2A	*snf4*Δ::*FRT*/*snf4*Δ::*FRT SNF1*^L181I^*-SAT1-FLIP*/*SNF1*^L181I^*-FRT*	This study
SCΔ*snf4*SNF1^L181I^M3B	SCΔ*snf4*SNF1^L181I^M2B	*snf4*Δ::*FRT*/*snf4*Δ::*FRT SNF1*^L181I^*-SAT1-FLIP*/*SNF1*^L181I^*-FRT*	This study
SCΔ*snf4*SNF1^L181I^M4A	SCΔ*snf4*SNF1^L181I^M3A	*snf4*Δ::*FRT*/*snf4*Δ::*FRT SNF1*^L181I^*-FRT*/*SNF1*^L181I^*-FRT*	This study
SCΔ*snf4*SNF1^L181I^M4B	SCΔ*snf4*SNF1^L181I^M3B	*snf4*Δ::*FRT*/*snf4*Δ::*FRT SNF1*^L181I^*-FRT*/*SNF1*^L181I^*-FRT*	This study
SCΔ*snf4*SNF1^ΔC310^M1A	SCSNF4M4A	*snf4*Δ::*FRT*/*snf4*Δ::*FRT SNF1*/*SNF1*^ΔC310^*-SAT1-FLIP*	This study
SCΔ*snf4*SNF1^ΔC310^M1B	SCSNF4M4B	*snf4*Δ::*FRT*/*snf4*Δ::*FRT SNF1*/*SNF1*^ΔC310^*-SAT1-FLIP*	This study
SCΔ*snf4*SNF1^ΔC310^M2A	SCΔ*snf4*SNF1^ΔC310^M1A	*snf4*Δ::*FRT*/*snf4*Δ::*FRT SNF1*/*SNF1*^ΔC310^*-FRT*	This study
SCΔ*snf4*SNF1^ΔC310^M2B	SCΔ*snf4*SNF1^ΔC310^M1B	*snf4*Δ::*FRT*/*snf4*Δ::*FRT SNF1*/*SNF1*^ΔC310^*-FRT*	This study
SCΔ*snf4*SNF1^ΔC340^M2A	SCSNF4M4A	*snf4*Δ::*FRT*/*snf4*Δ::*FRT SNF1*/*SNF1*^ΔC340^*-FRT*	19
SCΔ*snf4*SNF1^ΔC340^M2B	SCSNF4M4B	*snf4*Δ::*FRT*/*snf4*Δ::*FRT SNF1*/*SNF1*^ΔC340^*-FRT*	19
SCΔ*kis1*SNF1^Δ311 − 316^M1A	SCKIS1M4A	*kis1*Δ::*FRT*/*kis1*Δ::*FRT SNF1*/*SNF1*^Δ311 − 316^*-SAT1-FLIP*	This study
SCΔ*kis1*SNF1^Δ311 − 316^M1B	SCKIS1M4B	*kis1*Δ::*FRT*/*kis1*Δ::*FRT SNF1*/*SNF1*^Δ311 − 316^*-SAT1-FLIP*	This study
SCΔ*kis1*SNF1^Δ311 − 316^M2A	SCΔ*kis1*SNF1^Δ311 − 316^M1A	*kis1*Δ::*FRT*/*kis1*Δ::*FRT SNF1*/*SNF1*^Δ311 − 316^*-FRT*	This study
SCΔ*kis1*SNF1^Δ311 − 316^M2B	SCΔ*kis1*SNF1^Δ311 − 316^M1B	*kis1*Δ::*FRT*/*kis1*Δ::*FRT SNF1*/*SNF1*^Δ311 − 316^*-FRT*	This study
SCΔ*kis1*SNF1^Δ311 − 316^M3A	SCΔ*kis1*SNF1^Δ311 − 316^M2A	*kis1*Δ::*FRT*/*kis1*Δ::*FRT SNF1*^Δ311 − 316^*-SAT1-FLIP*/*SNF1*^Δ311 − 316^*-FRT*	This study
SCΔ*kis1*SNF1^Δ311 − 316^M3B	SCΔ*kis1*SNF1^Δ311 − 316^M2B	*kis1*Δ::*FRT*/*kis1*Δ::*FRT SNF1*^Δ311 − 316^*-SAT1-FLIP*/*SNF1*^Δ311 − 316^*-FRT*	This study
SCΔ*kis1*SNF1^Δ311 − 316^M4A	SCΔ*kis1*SNF1^Δ311 − 316^M3A	*kis1*Δ::*FRT*/*kis1*Δ::*FRT SNF1*^Δ311 − 316^*-FRT*/*SNF1*^Δ311 − 316^*-FRT*	This study
SCΔ*kis1*SNF1^Δ311 − 316^M4B	SCΔ*kis1*SNF1^Δ311 − 316^M3B	*kis1*Δ::*FRT*/*kis1*Δ::*FRT SNF1*^Δ311 − 316^*-FRT*/*SNF1*^Δ311 − 316^*-FRT*	This study
SCSNF1^Δ311 − 316^M1A and -B	SC5314	*SNF1*/*SNF1*^Δ311 − 316^*-SAT1-FLIP*	This study
SCSNF1^Δ311 − 316^M2A	SCSNF1^Δ311 − 316^M1A	*SNF1*/*SNF1*^Δ311 − 316^*-FRT*	This study
SCSNF1^Δ311 − 316^M2B	SCSNF1^Δ311 − 316^M1B	*SNF1*/*SNF1*^Δ311 − 316^*-FRT*	This study
SCSNF1^Δ311 − 316^M3A	SCSNF1^Δ311 − 316^M2A	*SNF1*^Δ311 − 316^*-SAT1-FLIP*/*SNF1*^Δ311 − 316^*-FRT*	This study
SCSNF1^Δ311 − 316^M3B	SCSNF1^Δ311 − 316^M2B	*SNF1*^Δ311 − 316^*-SAT1-FLIP*/*SNF1*^Δ311 − 316^*-FRT*	This study
SCSNF1^Δ311 − 316^M4A	SCSNF1^Δ311 − 316^M3A	*SNF1*^Δ311 − 316^*-FRT*/*SNF1*^Δ311 − 316^*-FRT*	This study
SCSNF1^Δ311 − 316^M4B	SCSNF1^Δ311 − 316^M3B	*SNF1*^Δ311 − 316^*-FRT*/*SNF1*^Δ311 − 316^*-FRT*	This study

a *SAT1-FLIP* denotes the *SAT1* flipper cassette; *FRT* is the FLP recombination target sequence, one copy of which remains in the genome after recycling of the *SAT1* flipper cassette.

### Sequencing of the *SNF1* alleles.

The *SNF1* alleles of the suppressor mutants SCΔ*snf4*SupB and SCΔ*snf4*SupC were amplified with primers SNF1.01 and SNF1.04, which bind in the *SNF1* upstream and downstream regions, respectively, and the PCR products directly sequenced using additional internal primers. All oligonucleotide primers used in this study are listed in Table 2.

**TABLE 2:** Primers used in this study

Primer	Sequence (5′–3′)^*a*^
SNF1.01	ATCCGAGCTCACACAAAAGACAAGAAC
SNF1.04	AAGTGGGCCCATTAAAATTGGTTGAATTTATTG
SNF1.05	AAGTCCGCGGATTAAAATTGGTTGAATTTATTG
SNF1.09^*b*^	GCCAGATTATTTGTTACCGCC
SNF1.10^*b*^	GGCGGTAACAAATAATCTGGC
SNF1d311-316.01^*b*^	TATGCCAGATTTATCCAAAAACAAGAATAGCAAG
SNF1d311-316.02^*b*^	GGATATCTGGCATATCTTGTTTAAACCATTCATC
SNF1d311-316.03	GGTTTAAACAAGATATGCCAGATTTATCCAAAAACAAGAATAGCAAG
SNF1d311-316.04	CTTGCTATTCTTGTTTTTGGATAAATCTGGCATATCTTGTTTAAACC
SNF1dC311.03	CTTACTTTATT**TTA**ATCTGGCATATCTTGTTTAAACCATTCATC
SNF1dC311.04	GATATGCCAGAT**TAA**AATAAAGTAAGTAAGTACTAGCTTAGATTGGATG
SNF1G51R.01^*b*^	AGAATAAGACGGTATCAAATTCTCAAGACATT
SNF1-GFP.01^*b*^	TCATGAGCTCAGCAATCGAAATCAAGTAATGAGATAT
SNF1in-seq.01^*b*^	GGCATCTTGTGGATCTCCT
SNF1in-seq.02^*b*^	GGCAATACTCTACAGCAGC

a The added SacI and SacII restriction sites are underlined; the stop codon introduced after *SNF1* codon 310 is highlighted in bold.

b Primer used for sequencing the *SNF1* alleles of *snf4*Δ suppressor mutants.

### Plasmid constructions.

A *SNF1* allele lacking codons 311 to 316 was generated by PCR in the following way. The upstream region and the 5′ part of the *SNF1* coding region were amplified with primers SNF1.01 and SNF1d311-316.03, and the 3′ part of the *SNF1* coding region and downstream sequences were amplified with primers SNF1d311-316.04 and SNF1.05. Primers SNF1d311-316.03 and SNF1d311-316.04 are complementary and lack *SNF1* codons 311 to 316. The gel-purified PCR products were then used as the templates in a fusion PCR with primers SNF1.01 and SNF1.05, and the SacI/SacII-digested PCR product was substituted for the *SNF1* upstream region in the *SNF1* deletion construct pSNF1M1 (19) to obtain pSNF1^Δ311 − 316^. To generate the *SNF1*^ΔC310^ allele, the upstream region and a part of the *SNF1* coding region were amplified with primers SNF1.01 and SNF1dC311.03, which introduced a TAA stop codon instead of the Tyr311 codon. The *SNF1* downstream region was amplified with primers SNF1dC311.04 and SNF1.05. The gel-purified PCR products served as the templates in a fusion PCR with primers SNF1.01 and SNF1.05, and the SacI/SacII-digested PCR product was substituted for the *SNF1* upstream region in the *SNF1* deletion construct, yielding pSNF1^ΔC310^.

### Strain constructions.

C. albicans strains were transformed by electroporation (32) with the gel-purified insertions from plasmids pSNF1^Δ311 − 316^, pSNF1^ΔC310^, pSNF1^ΔC340^ (19), and pSNF1^L181I^ (19). The correct genomic integration of all constructs and excision of the *SAT1* flipper cassette were confirmed by Southern hybridization using the flanking sequences as probes.

### Isolation of genomic DNA and Southern hybridization.

Genomic DNA from C. albicans strains was isolated as described previously (33). The DNA was digested with appropriate restriction enzymes, separated on a 1% agarose gel, transferred by vacuum blotting onto a nylon membrane, and fixed by UV cross-linking. Southern hybridization with enhanced chemiluminescence-labeled probes was performed with an Amersham ECL direct nucleic acid labeling and detection system (GE Healthcare UK Limited, Little Chalfont Buckinghamshire, United Kingdom) according to the instructions of the manufacturer.

### Phenotypic assays.

Growth on different carbon sources and resistance to cell wall/membrane stress were tested by dilution spot assays as described in the legends to the figures. Carbon source utilization was tested on YNB agar plates (0.67% yeast nitrogen base with ammonium sulfate, 2% agar) containing 2% glucose, sucrose, potassium acetate, or glycerol. Growth on sucrose was also tested on YPD plates containing 2% sucrose instead of glucose (YPS). Cell wall/membrane stress resistance was tested on YPD plates containing 50 μg/ml Congo red, 15 mM caffeine, or 0.04% SDS. Growth curves in liquid media were obtained by cultivating the strains in microtiter plates and measuring the OD of the cultures every 10 min in a Tecan Infinite F200 PRO plate reader.

### Western blotting.

Overnight cultures of the strains were diluted 10^−2^ in 50 ml fresh YPD medium and grown for 3 h at 30°C. For growth on alternative carbon sources, the strains were incubated for only 1 h at 30°C, washed in sterile water, resuspended in 50 ml of YP medium supplemented with 2% of the different carbon sources, and grown for additional 2 h. Cells were collected by centrifugation, washed in 50 ml H_2_O, and resuspended in 500 μl breaking buffer (100 mM triethylammonium bicarbonate buffer [TEAB], 150 mM NaCl, 1% SDS, cOmplete EDTA-free Protease Inhibitor Cocktail, and PhosStop Phosphatase Inhibitor Cocktail [Roche Diagnostics GmbH, Mannheim, Germany]) supplemented with protease and phosphatase inhibitors. Equal volumes of 0.5-mm-diameter acid-washed glass beads were added to all tubes. Cells were mechanically disrupted on a FastPrep-24 cell homogenizer (MP Biomedicals, Santa Ana, CA, USA) with three 40-s pulses, with 5 min on ice between pulses. Samples were centrifuged at 13,000 rpm for 15 min at 4°C, the supernatant was collected, and the protein concentration was quantified using the Bradford protein assay. Equal amounts of protein of each sample were mixed with 1 volume of 2× Laemmli buffer, heated for 5 min at 95°C, and separated on an SDS-9% polyacrylamide gel. Separated proteins were transferred onto a nitrocellulose membrane with a mini-Protean system (Bio-Rad, Munich, Germany) and stained with Ponceau S to control for equal loading. To detect T208 phosphorylation of Snf1, membranes were blocked in 5% BSA–TBST (5% bovine serum albumin–Tris-buffered saline with Tween 20) at room temperature for 1 h and subsequently incubated overnight at 4°C with Phospho-AMPKα (Thr172) antibody (catalog no. 2531; Cell Signaling Technology, Danvers, MA, USA). Membranes were washed in TBST and incubated at room temperature for 1 h with anti-rabbit horseradish peroxidase (HRP) G-21234 antibody (Invitrogen GmbH, Darmstadt, Germany).
